# Predicting the Risk Genes of Autism Spectrum Disorders

**DOI:** 10.3389/fgene.2021.665469

**Published:** 2021-06-14

**Authors:** Yenching Lin, Srinivasulu Yerukala Sathipati, Shinn-Ying Ho

**Affiliations:** ^1^Interdisciplinary Neuroscience Ph.D. Program, National Chiao Tung University, Hsinchu, Taiwan; ^2^Center for Precision Medicine Research, Marshfield Clinic Research Institute, Marshfield, WI, United States; ^3^Institute of Bioinformatics and Systems Biology, National Chiao Tung University, Hsinchu, Taiwan; ^4^Institute of Population Health Sciences, National Health Research Institutes, Miaoli, Taiwan; ^5^Institute of Bioinformatics and Systems Biology, National Yang Ming Chiao Tung University, Hsinchu, Taiwan; ^6^Department of Biological Science and Technology, National Yang Ming Chiao Tung University, Hsinchu, Taiwan; ^7^Center For Intelligent Drug Systems and Smart Bio-Devices (IDS^2^B), National Chiao Tung University, Hsinchu, Taiwan

**Keywords:** autism spectrum disorders, gene expression profiles, machine learning, risk gene prediction, feature selection

## Abstract

Autism spectrum disorder (ASD) refers to a wide spectrum of neurodevelopmental disorders that emerge during infancy and continue throughout a lifespan. Although substantial efforts have been made to develop therapeutic approaches, core symptoms persist lifelong in ASD patients. Identifying the brain temporospatial regions where the risk genes are expressed in ASD patients may help to improve the therapeutic strategies. Accordingly, this work aims to predict the risk genes of ASD and identify the temporospatial regions of the brain structures at different developmental time points for exploring the specificity of ASD gene expression in the brain that would help in possible ASD detection in the future. A dataset consisting of 13 developmental stages ranging from 8 weeks post-conception to 8 years from 26 brain structures was retrieved from the BrainSpan atlas. This work proposes a support vector machine–based risk gene prediction method ASD-Risk to distinguish the risk genes of ASD and non-ASD genes. ASD-Risk used an optimal feature selection algorithm called inheritable bi-objective combinatorial genetic algorithm to identify the brain temporospatial regions for prediction of the risk genes of ASD. ASD-Risk achieved a 10-fold cross-validation accuracy, sensitivity, specificity, area under a receiver operating characteristic curve, and a test accuracy of 81.83%, 0.84, 0.79, 0.84, and 72.27%, respectively. We prioritized the temporospatial features according to their contribution to the prediction accuracy. The top identified temporospatial regions of the brain for risk gene prediction included the posteroventral parietal cortex at 13 post-conception weeks feature. The identified temporospatial features would help to explore the risk genes that are specifically expressed in different brain regions of ASD patients.

## Introduction

Autism spectrum disorder (ASD) is a neurodevelopmental disorder characterized by a repetitive behavior, difficulty in communication, and deceit in social interaction. A variety of psychiatric symptoms such as depression, aggression, and Tourette disorders are also observed among adults with ASD (Ghaziuddin et al., 1995; Green et al., 2000; Sverd, 2003). The prevalence of ASD is estimated to be 2 or more in 1,000 children (Newschaffer et al., 2007) and 14% among siblings of female with autistic disorder (Ritvo et al., 1989). There are some debates on genetics and environmental factors that influence ASD. For example, there is an emerging evidence that demonstrated that heritability is one of the important factors that associated with ASD. Genomic variations such as genetic syndromes, copy number variations, and mutations were observed in approximately 20% of the cases with ASD (Abrahams and Geschwind, 2008; Rosenberg et al., 2009). In contrast, a twin study reported that environmental liability also influences the ASD risk (Nordenbæk et al., 2014). A large population-based study on siblings including monozygotic and dizygotic twins reported that equal contribution of environmental factors and hereditary are the important risk factors of ASD (Sandin et al., 2014). However, some evidences reported that genetics contribute more to the ASD etiology than environmental factors, and recent progress in genetic discovery improved better the understanding of the etiology of ASD (Kim and Leventhal, 2015). The genetic etiology of ASD and related neurobiological mechanisms at many levels ranging from molecules to circuits were acknowledged in various studies (Hicks and Middleton, 2016). Basu et al. (2009) reported a list of 1,237 human genes that have potential connections to ASD. GeneCards^1^ presents a prioritized list of 7,207 genes related to autism. Currently, only about 65 genes out of an estimated several hundred are known to be involved in ASD based on strong genetic evidences (Sanders et al., 2015).

Numerous genes are reported in the ASD phenotype. For instance, neuromedin U involves in the modulation of dopaminergic actions (Raddatz et al., 2000), and GBX2 regulates midbrain and cellular development (Waters and Lewandoski, 2006). Human serotonin receptor 2B and CENTG2 have also been considered as important candidates for ASD (Lin et al., 2004; Lukusa et al., 2004). There are several studies that demonstrated the involvement of genetic variants in ASD (Buxbaum et al., 2002; Kim et al., 2006). For instance, single nucleotide polymorphism in Gamma-aminobutyric acid A receptor beta 3 (GABRB3) is involved in gene expression, genome instability, and recombination and is also significantly associated with ASD (Buxbaum et al., 2002; Kim et al., 2006). The oxytocin receptor gene modulates the cognition and communication abilities in individuals diagnosed with ASD (Lerer et al., 2008). Genome-wide association studies that focused on the genetic basis of psychiatric disorders reported the common genetic variants in ASD (Ma et al., 2009). Mutations in synaptic genes such as neuroligins and neurexin families are consistently observed in ASD (Jamain et al., 2003; Graf et al., 2004). Post-transcriptional mechanism, such as miRNA that broadly influences gene expression without altering the DNA code, represents one means of altering the entire gene networks (Fregeac et al., 2016). There are some attempts to describe the spatiotemporal gene expression patterns. Ramirez-Celis et al. (2021) identified the presence of maternal autoantibodies to fetal brain proteins specific to ASD. This study has validated a serological assay to identify ASD-specific maternal autoantibody patterns of reactivity against eight proteins (CRMP1, CRMP2, GDA, NSE, LDHA, LDHB, STIP1, and YBOX) that are highly expressed in developing brain, and determine the relationship of these reactivity patterns with ASD outcome severity.

A novel class of regulatory RNAs, long noncoding RNAs (lncRNAs) are emerging as important post-transcriptional regulators in a number of fundamental gene regulatory events, but their role in autism disorders remains unknown. LncRNAs (Ziats and Rennert, 2013), defined as RNAs greater than 200 nucleotides in length, have been shown to be involved in major mechanisms of gene expression regulations, such as targeting transcription factors, initiating chromatin remodeling, directing methylation complexes, and blocking nearby transcription (Ponting et al., 2009). Moreover, pervasive transcription of lncRNAs has been found to be involved during development process (Amaral and Mattick, 2008). Accumulating evidences show that lncRNAs are implicated in ASD risk (Wilkinson and Campbell, 2013; Zhang et al., 2019). Due to the fact that the genetic diagnosis of ASD depends on multiple genetic markers, current genetic diagnostic methods are inadequate for clinical utility and applications. Additionally, gene identification studies are laborious and cost effective. Hence, prediction methods are necessary to identify multiple genetic markers that provide useful information for early stage detection and ASD diagnosis.

Machine learning methods have been used to identify the genetic markers to diagnose ASD. Machine learning–based studies have already been attempted to prioritize the high-confidence gene candidates by constructing cell type-specific predictive models that can promote the diagnosis of ASD (Guan et al., 2020). Trifonova et al. (2019) prioritized the genes cataloged in Simon’s Foundation Autism Research Initiative (SFARI) database, and gene network analysis revealed that 79% of the genes from SFARI were connected to the mechanistic target of rapamycin-modulated genes. Different machine learning and deep learning approaches were developed to predict the candidate lncRNAs associated with ASD (Wang and Wang, 2020). Skafidas et al. (2014) used SNP data to create a genetic diagnostic classifier to predict ASD diagnosis and obtained a good accuracy in homogenous population. Hu and Lai (2013) utilized gene expression signature from lymphoblastoid cell lines and support vector machine (SVM) for the identification of genes that are associated with autism. Structural brain gender differences in brain structures were identified using an SVM classifier (Retico et al., 2016), and increased gray matter in young children with ASD was observed. The neuroanatomical networks involved in ASD were classified using SVM based on gray matter scans (Ecker et al., 2010). Cogill and Wang (2016) developed an SVM-based model and distinguished ASD risk genes with an accuracy of 76.7% and further prioritized the genes responsible for neurodevelopmental disorders. However, identifying the temporospatial regions of the brain regions that are associated with ASD is necessary to understand the etiology.

**FIGURE 1:**
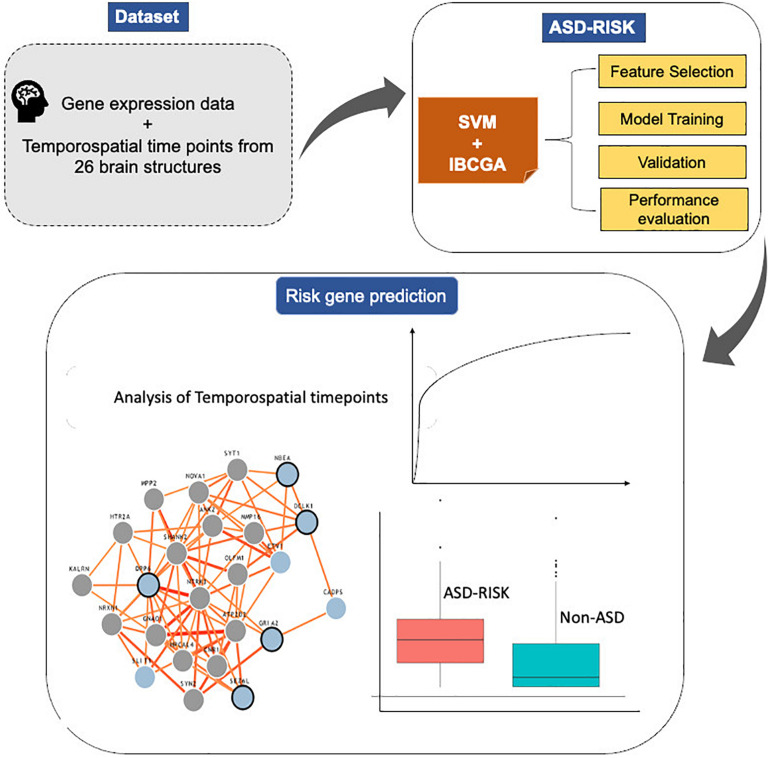
System flowchart of the proposed ASD-Risk.

The main objective of this work is to predict the risk genes of ASD and simultaneously select the important features that increase the prediction performance. This study proposed an SVM-based classifier, ASD-Risk, to categorize the risk genes of ASD and identify the temporospatial regions of the brain using gene expression profiles that are implicated in ASD. ASD-Risk used an inheritable bi-objective combinatorial genetic algorithm (IBCGA) (Ho et al., 2004b) to select a small set of temporospatial features from various developmental time points from 26 brain structures. A dataset consisting of 732 gene expression profiles across 13 developmental stages ranging from 8 weeks post-conception to 8 years from 26 brain structures was retrieved from the BrainSpan atlas database and previous work (Cogill and Wang, 2016). ASD-Risk identified 19 temporospatial regions and time points that are significantly associated with the risk genes of ASD and non-ASD and achieved a 10-fold cross-validation (10-CV) accuracy, sensitivity, specificity, area under the curve (AUC), and test accuracy of 81.83%, 0.84, 0.79, 0.84, and 72.27%, respectively. We compared the prediction performance of ASD-Risk with a previous work (Cogill and Wang, 2016) and some standard machine learning methods. Next, the identified 19 temporospatial features were ranked based on their contribution toward the prediction performance. The top 10 ranked temporospatial features were analyzed further. The system flowchart of this work is shown in Figure 1.

## Results

### Evaluation and Performance Comparison of ASD-Risk

We used a dataset consisting of 732 samples including 366 risk genes of ASD and 366 disease genes (non-ASD) expressed across different developmental stages and time points of 26 brain structures. ASD-Risk was incorporated with a feature selection algorithm IBCGA to select a small set of temporospatial features associated with the risk genes of ASD. ASD-Risk obtained a 10-CV accuracy, sensitivity, specificity, Mathew correlation coefficient (MCC), AUC, and test accuracy of 81.83%, 0.84, 0.79, 0.63, 0.84, and 72.27%, respectively.

The major objective of ASD-Risk is to identify the temporospatial regions that are associated with the risk genes of ASD and accurately predict the risk genes of ASD and non-ASD. ASD-Risk identified 19 temporospatial regions and time points of brain structures and effectively distinguished the risk genes of ASD and non-ASD. Furthermore, prediction performance of ASD-Risk was compared with some standard machine learning methods of Waikato Environment for Knowledge Analysis (Weka) datamining software (Frank et al., 2004). Machine learning methods, including random forest (RF), logistic model tree (LMT), sequential minimal optimization (SMO), and simple logistic method, were used for performance comparison. The performance of ASD-Risk achieved a 10-CV accuracy, sensitivity, specificity, and MCC of 81.83%, 0.84, 0.79, and 0.63, respectively, whereas RF achieved 72%, 0.69, 0.66, and 0.44, respectively, LMT achieved 73%, 0.81, 0.71, and 0.45, respectively; SMO achieved 74%, 0.77, 0.76, and 0.48, respectively, and simple logistic method achieved 73%, 0.81, 0.71, and 0.46, respectively. The prediction performance of ASD-Risk is better than that of some machine learning methods. ASD-Risk on a full training dataset obtained a 10-CV, sensitivity, specificity, AUC, and MCC of 80.05%, 0.81, 0.78, 0.81, and 0.60, respectively. The performance comparison results are shown in Table 1. ASD-Risk identified 19 temporospatial regions of the developmental time points, which can efficiently distinguish risk genes of ASD from non-ASD genes and are listed in Table 2.

**TABLE 1:** Performance comparison among ASD-Risk and some typical classifiers.

**Method**	**10-ACC (%)**	**Sensitivity**	**Specificity**	**MCC**	**Test ACC (%)**
Random forest	72.0	0.69	0.66	0.44	68.0
LMT	73.0	0.81	0.71	0.45	73.0
SMO	74.0	0.77	0.76	0.48	76.0
Simple logistic	73.0	0.81	0.71	0.46	74.0
ASD-Risk	81.8	0.84	0.79	0.63	72.2

**TABLE 2:** MED and feature knockout analysis of identified brain structural and time points in ASD.

**Rank**	**Features**	**Structure**	**Time point**	**MED Score**	**Performance difference (%)**
1	IPC_13pcw_F_12834	Posteroventral (inferior) parietal cortex	13 pcw	23.63	6.43
2	V1C_8yrs_M_12841	Primary visual cortex (striate cortex area V1/17)	8 years	23.24	7.79
3	STC_16pcw_M_12837	Posterior superior temporal cortex (area S1, area 3,1,2)	16 pcw	18.16	6.56
4	STR_13pcw_M_12820	Striatum	13 pcw	14.64	7.24
5	OFC_40yrs_F_12304	Orbital frontal cortex	40 years	14.64	6.43
6	MFC_8pcw_M_13058	Anterior (rostral) cingulate (medial prefrontal) cortex	8 pcw	11.91	6.7
7	DTH_12pcw_F_12960	Dorsal thalamus	12 pcw	11.52	6.56
8	AMY_8yrs_M_12981	Amygdaloid complex	8 years	10.35	6.56
9	A1C_11yrs_F_12289	Primary auditory cortex (core)	8 years	10.35	6.43
10	HIP_21yrs_F_13057	Hippocampus (hippocampal formation)	11 years	8.00	6.29
11	V1C_1yrs_F_12830	Primary visual cortex (striate cortex area V1/17)	1 year	7.61	6.97
12	ITC_11yrs_F_12289	Inferolateral temporal cortex (area TEv, area 20)	11 years	6.83	6.56
13	STC_1yrs_F_12830	Posterior superior temporal cortex (area S1, area 3,1,2)	1 year	4.49	6.15
14	A1C_16pcw_M_12837	Primary auditory cortex (core)	16 pcw	4.10	6.29
15	MD_37yrs_M_12303	Mediodorsal nucleus of thalamus	37 years	4.10	7.11
16	MFC_9pcw_M_12833	Anterior (rostal) cingulate (medial prefrontal) cortex	9 pcw	3.32	6.15
17	HIP_21pcw_M_12886	Hippocampus (hippocampal formation)	21 pcw	3.32	7.24
18	ITC_13pcw_M_12820	Inferolateral temporal cortex (area TEv, area 20)	13 pcw	2.14	6.15
19	DFC_11yrs_F_12289	Dorsolateral prefrontal cortex	11 years	2.14	6.7

Additionally, the prediction performance of ASD-Risk was compared with the prediction model proposed by Cogill and Wang (2016). ASD-Risk achieved a mean training accuracy, mean specificity, and mean sensitivity of 86.03 ± 0.86, 0.92 ± 0.01, and 0.52 ± 0.01, respectively, while the SVM model proposed by Cogill and Wang achieved a mean training accuracy, mean specificity, and mean sensitivity of 76.7, 77.2, and 74.4%, respectively. ASD-Risk performed better than the previous method in terms of mean training accuracy and specificity.

### Ranks of the Temporospatial Features

We ranked the identified temporospatial features according to their contribution to the ASD risk gene prediction using main effect difference (MED) analysis (Tung and Ho, 2008). According to the MED analysis, the feature with the highest rank contributed more toward the risk gene prediction. The top 10 ranked temporospatial regions of the brain structures include the posteroventral parietal cortex at 13 post-conception weeks (pcw), the primary visual cortex at 8 years, the posterior superior temporal cortex at 16 pcw, the striatum at 13 pcw, the orbital frontal cortex at 40 years, the anterior cingulate (medial prefrontal) cortex at 8 pcw, the dorsal thalamus at 12 pcw, the amygdaloid complex at 8 years, the primary auditory cortex at 8 years, and the hippocampus at 11 years. The identified temporospatial features, corresponding ranks, and MED scores are given in Table 2. The selected features were described by the time points and the brain structure where the samples were collected.

### ASD Risk Genes Expressed in Brain Regions

Furthermore, we analyzed the importance of the top 10 ranked temporospatial features in ASD as follows.

**(1) Posteroventral (inferior) parietal cortex—13 pcw:** The age of developing embryo or fetus is often described in terms of pcw. A study on autistic children and their families reported that regional perfusion variations are observed in the parietal cortex of autistic children and their first-degree family members (Degirmenci et al., 2008). Raphael et al. reported that mutations in chromodomain helicase DNA-binding protein 8 (CHD8) were associated with ASD diagnosis; CHD8 is expressed in the fetal brain region of the posteroventral (inferior) parietal cortex (Bernier et al., 2014). A genome-wide association study observed a significant enrichment in the expressions of individual genes PANX1, SLC25A12, and PANX2 at quantitative trait loci in the parietal cortex that is implicated in autism disorder (Davis et al., 2012). Furthermore, we analyzed the genetic changes in brain regions and developmental stages that are linked to ASD using genome-wide prediction of the autism associated gene database (Krishnan et al., 2016). Gene expression signatures specific to the posteroventral (inferior) parietal cortex at early mid-fetal were Pumilio RNA-binding family member 2, guanine nucleotide binding protein (G protein), alpha activating activity polypeptide O, Nipped-B homolog (Drosophila), bromodomain PHD finger transcription factor, and K (lysine) acetyltransferase 6A.

**(2) Primary visual cortex (striate cortex area V1/17)—8 years:** Casanova et al. (2006) investigated a set of postmortem brains of individuals with ASD and observed a greater number of cells per minicolumn in the primary sensory and visual cortexes when compared to the brain samples of controls. A genomic analysis study on the coexpression gene network of ASD observed that ASD genes regulate different biological functions during human cortical development (Parikshak et al., 2013). Protein-altering rare *de novo* variation-affecting genes with high connectivity in the primary visual cortex were JMJD1C, RBM27, PPM1D, CNOT6, and MLL3 (Parikshak et al., 2013). Genome-wide prediction analysis results revealed that five ASD genes specific to the primary visual cortex at middle late childhood were ATPase, Ca2+ transporting, plasma membrane 2, sema domain, transmembrane domain (TM), and cytoplasmic domain, (semaphorin) 6D, Synaptosomal-associated protein, 25 kDa, zinc finger protein 148, and proteasome (prosome, macropain) subunit, alpha type 1.

**(3) Posterior superior temporal cortex (area S1, area 3, 1, 2)—16 pcw:** The posterior superior temporal cortex is part of the temporal lobe including Broca’s motor speech area and Wernicke’s area. The important role of the posterior superior temporal cortex is to integrate lexical-semantic and syntactic information during sentence comprehension (Friederici et al., 2009). A systematic analysis of the expression of molecular markers in postmortem brain samples from children with autism observed that focal disruptions of acritical laminar architecture were identified in the posterior superior temporal cortex of young children with autism (Stoner et al., 2014). The predicted ASD genes that are enriched in the superior temporal cortex at early fetal include pumilio RNA-binding family member 2, Nipped-B homolog (Drosophila), bromodomain PHD finger transcription factor, K(lysine) acetyltransferase 6A, and B-cell CLL/lymphoma 11A (zinc finger protein).

**(4) Striatum-13pcw:** Fuccillo and co-authors investigated multiple genetic mouse models of ASD to identify the abnormalities in striatal circuits that constitute a common pathophysiological mechanism in the development of autism-related behaviors, and reported that striatal dysfunction is intimately associated with the etiology and pathophysiology of ASD (Fuccillo, 2016). A gene expression analysis study observed a highly restricted pattern of expression in contactin associated protein-like 2, a member of the neurexin family in the striatum and frontal and anterior temporal lobes (Alarcón et al., 2008). Araujo et al. (2015) reported that fork head box p1 protein levels substantially reduced by approximately 50% in the stratum relevant to ASD. The preferential gene mutations in ASD were often reported in the striatum region (Shohat et al., 2017). The ASD gene signature of stratum regions consist of the sema domain, transmembrane domain (TM), cytoplasmic domain, (semaphorin) 6D, pumilio RNA-binding family member 1, guanine nucleotide binding protein (G protein), beta polypeptide 1, RING1 and YY1 binding protein, K (lysine) acetyltransferase 6A, and runt-related transcription factor 1, translocated to 1 (cyclin D-related).

**(5) Orbital frontal cortex—40 years:** Hu et al. (2015) reported that the expression of retinoic acid-related orphan receptor alpha was highly correlated with the NLGN1 gene in the orbital frontal cortex of female adults with ASD. Genome-wide prediction analysis results reported that the genes specific to the orbital frontal cortex-early mid-fetal 2 were spen homolog, transcriptional regulator (Drosophila), runt-related transcription factor 1, translocated to 1 (cyclin D-related), pumilio RNA-binding family member 1, K(lysine) acetyltransferase 6A, and AF4/FMR2 family, member 2.

**(6) Anterior (rostral) cingulate (medial prefrontal) cortex—8 pcw:** Chandley and co-authors reported that the expression alterations of genes SLC1A1, GRIN1, GRIP1, and GRM8 were observed in the neocortical pyramidal neurons of the anterior cingulate cortex. The reduced NTRK2 expression was observed in the anterior cingulate cortex of individuals with ASD (Chandley et al., 2015). The predicted genes specific to the medial prefrontal cortex-neonatal-early infancy were arginine-glutamic acid dipeptide (RE) repeats, latrophilin 1, catenin (cadherin-associated protein), delta 2, chondroitin sulfate proteoglycan 5 (neuroglycan C), and Rho GDP dissociation inhibitor (GDI) alpha.

**(7) Dorsal thalamus—12 pcw:** A gene expression analysis study on ASD reported that higher expression levels of CNTNAP2 were observed in the dorsal thalamus of a 19-week fetal brain (Alarcón et al., 2008). A high level expression pattern of autism susceptibility candidate 2 was detected in dorsal thalamus regions in patients with ASD (Bedogni et al., 2010). The predicted genes specific to the mediodorsal nucleus of the thalamus-early mid-fetal were synapsin II, RING1 and YY1 binding protein, neurexin 1, ankyrin 2, and neuronal and AF4/FMR2 family member 2.

**(8) Amygdaloid complex—8 years:** A microarray study reported that Homer1a is significantly upregulated in the amygdala and altered the function of ASD-related proteins such as metabotropic glutamate receptors and Shank3 (Banerjee et al., 2016). A magnetic resonance spectroscopic study demonstrated the higher concentrations of glutamate/glutamine observed in the amygdala-hippocampal region of individuals with ASD (Page et al., 2006). The predicted ASD genes specific to the amygdala complex-middle late childhood were synapsin II, slit homolog 1 (Drosophila), pleckstrin, and Sec7 domain containing 3,5-hydroxytryptamine (serotonin) receptor 2C, G protein-coupled and glutamate receptor, ionotropic, and AMPA 2.

**(9) Primary auditory cortex (core)—8 years:** Xiong et al. (2012) reported that autism candidate genes, phosphatase and tensin homolog, were found to be deleted on chromosome 10, and these genes were implicated in the primary auditory cortex of mouse models. The predicted ASD genes specific to the primary auditory cortex-young adulthood were trinucleotide repeat containing 6B, neurofascin, fragile X mental retardation 1, the connector enhancer of the kinase suppressor of Ras 2, and calmodulin binding transcription activator 1.

**FIGURE 2:**
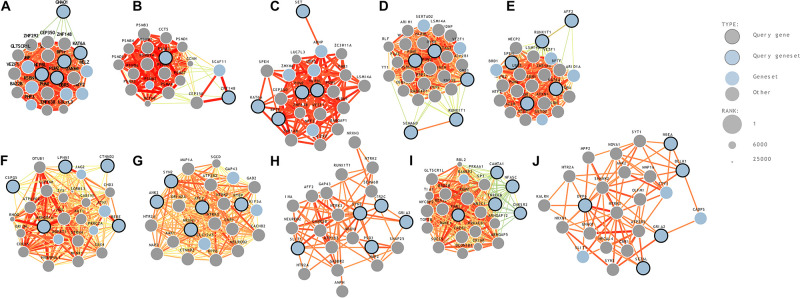
Genome-wide prediction of ASD-associated genes specific to temporospatial features. **(A)** posteroventral parietal cortex, **(B)** primary visual cortex, **(C)** posterior superior temporal cortex, **(D)** striatum, **(E)** orbital frontal cortex, **(F)** anterior cingulate cortex, **(G)** dorsal thalamus, **(H)** amygdaloid complex, **(I)** primary auditory cortex, and **(J)** hippocampus.

**(10) Hippocampus (hippocampal formation)—11 years:** A comparative gene expression analysis study on mouse models reported that two genes, BTBR and En2, were differentially expressed in the hippocampal region of ASD (Provenzano et al., 2016). A strong association between hippocampus and ASD in mouse models has previously been reported (Nadler et al., 2006). ASD genes predicted to be in the hippocampus region-middle late childhood were seizure-related 6 homolog (mouse)-like, neurobeachin, glutamate receptor, ionotropic, AMPA 2, dipeptidyl-peptidase 6, and doublecortin-like kinase 1. The genome-wide predictions of ASD-associated genes specific to different brain regions and time points are shown in Figure 2. The risk gene expression levels at different brain regions that are linked to ASD can be accessed from the genome-wide prediction of the autism-associated gene database (Krishnan et al., 2016).

Additionally, we employed the feature knockout analysis to investigate the individual feature contribution to the prediction accuracy. The feature knockout analysis revealed that removal of each feature reduced the prediction performance on an average of 6.64 ± 0.44. The performance difference showed that the identified features were potential candidates to distinguish the risk genes of ASD and non-ASD genes. The feature knockout analysis results are shown in Table 2.

**FIGURE 3:**
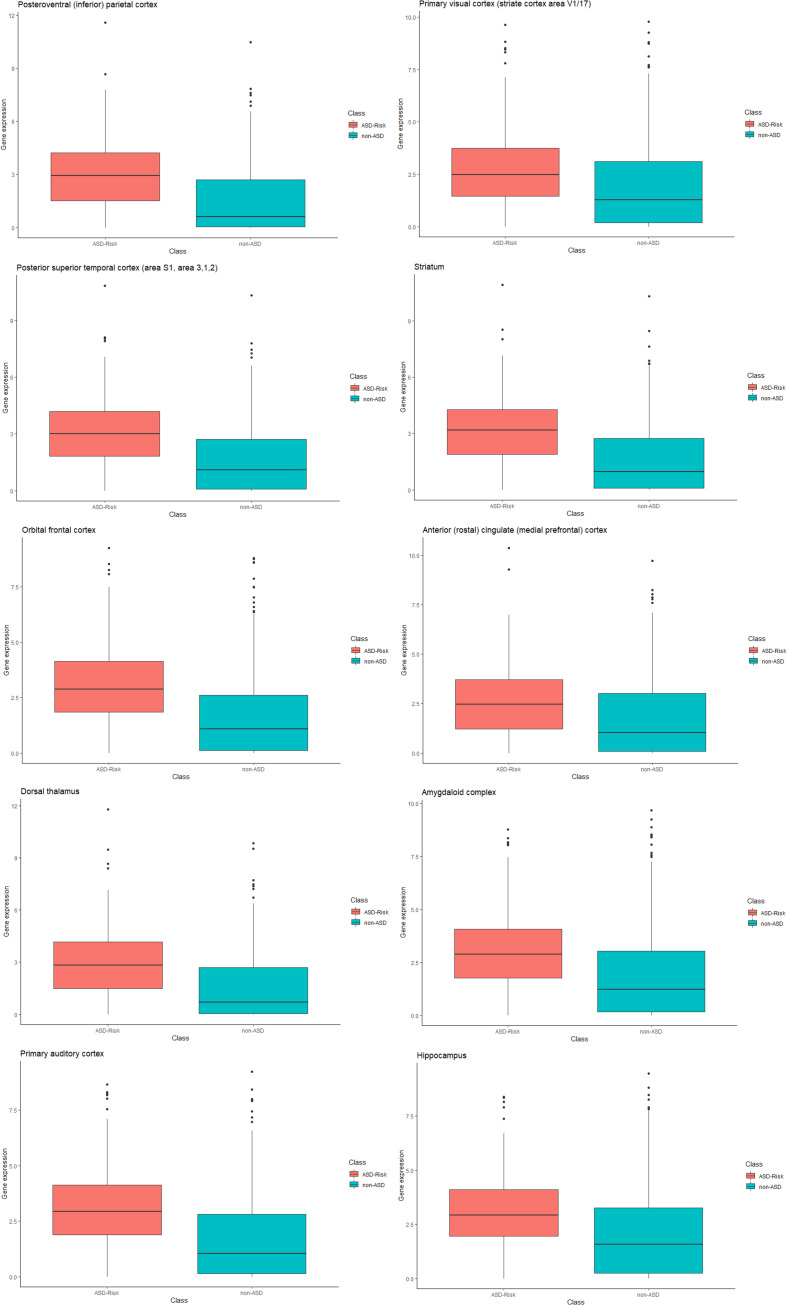
Box plot representation of gene expression in ASD and non-ASD. Each box plot represents gene expression differences in temporospatial time points. The X-axis represents ASD and non-ASD expression levels across all samples, and the Y-axis represents gene expression values (RPKM).

Furthermore, we compared the relative gene expression levels between the risk genes of ASD and non-ASD genes. The significant difference was observed between the expression levels of the risk genes of ASD and non-ASD genes. Differences in the expression level of genes at temporospatial regions between the ASD and non-ASD are shown in Figure 3 using box plot analysis.

## Conclusion

Identifying the brain temporospatial regions where the risk genes are expressed in ASD patients is necessary to understand the genetic variations in ASD etiology and early diagnosis. ASD shares behavioral characteristics and symptoms with other neurological disorders. Hence, exploring genetic variations in ASD also provides the key information into other neurodevelopmental defects.

The objective of this work is identifying the important temporospatial regions and developmental timepoints of brain structures that can accurately predict the risk genes of ASD. In this work, ASD-Risk used an optimal feature selection algorithm IBCGA to identify 19 brain temporospatial regions to classify the risk genes of ASD and non-ASD. ASD-Risk obtained a 10-CV accuracy, sensitivity, specificity, AUC, and test accuracies of 81.83, 72.27%, 0.84, 0.79, and 0.84, respectively. The prediction performance of ASD-Risk was comparatively better than other machine learning methods. Furthermore, the identified top 10 ranked temporospatial regions revealed their roles in ASD etiology. The neurulation, neurogenesis, neuronal migration, and formation of cortical cell layers are critical events in normal fetal brain development since post-conception weeks 8–20 (Linderkamp et al., 2009). Five of the top 10 ranked temporospatial regions and developmental time points including the posteroventral (inferior) parietal cortex—13 pcw; the posterior superior temporal cortex (area S1, area 3,1,2)—16 pcw; the striatum—13 pcw; the anterior (rostral) cingulate (medial prefrontal) cortex—8 pcw; and the dorsal thalamus—12 pcw, were involved in fetal brain development stages. The pervasive transcription of lncRNAs has been demonstrated in a temporally and spatially regulated manner to differentiate between ASD and normal subjects during neural development. It allows suggesting that lncRNAs coordinate the genetic operation of neuron communication in the cortical network and limbic system, which play key roles in attention, planning, social interaction, and thought. This analysis identified temporospatial regions where the risk genes are expressed in ASD. This study would help to develop new genetic approaches implicated in neurodevelopment disorders.

## Methods

### Dataset

We downloaded the dataset consisting of 336 positive ASD risk gene and 1762 non-ASD disease genes from Cogill and Wang (2016). These data were generated across 13 developmental stages from 8 weeks post-conception to 40 years of age from 26 brain structures. The expressions of RNA-sequencing were read in the units of reads per kilobase of transcript per million mapped reads (RPKM), and aligned using the GENCODE consortium’s annotation release v10 (Harrow et al., 2012).

Cogill and Wang studied the brain gene expression data with 336 positive ASD risk gene and 1,762 non-ASD disease genes for prioritizing the autism risk gene candidates. The positive instances were derived by picking up the top 85% of the genes based upon the expression variance within the BrainSpan dataset as ASD risk genes compiled from the Simons Foundation Autism Research Initiative Gene database (Xu et al., 2012; De Rubeis et al., 2014). The genes associated with diseases but unrelated to the intellectual disability (ID) have been used as negative controls. We followed the same procedure to divide the positive and negative instances. The list of gene IDs and their expression values for the temporospatial timepoints and the risk gene information can be accessed from Cogill and Wang’s (2016) study. In the dataset, genes were instances and temporospatial regions, and time points were features for the training and test sets. In this work, we keep 336 positive ASD risk genes and randomly selected 336 non-ASD genes as a new balance dataset.

### ASD-Risk Model Formulation

Support vector machines (SVMs) are statistical learning algorithms that are explicitly used in solving many biological problems (Vapnik, 1999; Srinivasulu et al., 2015). In this study, we incorporated an optimal feature selection algorithm IBCGA and SVM to build the ASD-Risk. An SVM works implicitly in the feature space by computing only the corresponding kernel *K*(*x*_*i*_, *x*_*j*_) between any two objects *x*_*i*_ and *x*_*j*_:

(1) $$K\,\left(x_{i},x_{j}\right)=\phi \,{\left(x_{i}\right)}^{\text{T}}\,\phi \,\left(x_{j}\right)$$

where Φ(x) is a mapping function.

### Inheritable Bi-Objective Combinatorial Genetic Algorithm

IBCGA was used to solve bi-objective combinatorial problems. The IBCGA uses an intelligent evolutionary algorithm (Ho et al., 2004a), which is good at deriving an optimized SVM with feature selection. The IBCGA has been successfully applied in solving several biological problems (Yerukala Sathipati et al., 2016, 2019; Yerukala Sathipati and Ho, 2017, 2018, 2020, 2021).

We used common genetic algorithm (GA) terms “GA-gene” and “GA-chromosome.” In this problem, “GA-chromosome” contains 524 binary genes. Two 4-bit “GA-genes” were utilized for tuning the C and γ of the SVM. So, this method encodes the parameter C in the 2^–7^ to 2^–8^ interval and 16 values of γ. Normalized and digitalized gene expressions were used as the input in the SVM classifier. Gene expressions corresponding to temporospatial time points were considered as features. Parameter tuning of ASD-Risk is as follows; the candidate feature range selected by the IBCGA is *r*_*begin*_ = 30 and *r*_*end*_ = 10. The steps involved in the IBCGA are as follows.

Step 1: (Evaluation) Evaluate the fitness value of all *individuals* using the fitness function that is the prediction accuracy in terms of 10-fold cross-validation.
Step 2: (Selection) Use the tournament selection method that selects the winner from two randomly selected *individuals* to generate a mating pool.
Step 3: (Crossover) Select two *parents* from the mating pool to perform orthogonal array crossover operation.
Step 4: (Mutation) Apply a conventional mutation operator to the randomly selected *individuals* in the new population. Mutation is not applied to the best *individuals* to prevent the best fitness value from deterioration.
Step 5: (Termination test) If the stopping condition for obtaining the solution is satisfied, then output the best *individual* as the solution. Otherwise, go to Step 2.
Step 6: (Inheritance) If *r* < *r*_*end*_, randomly change one bit in the binary “GA-genes” for each *individual* from 0 to 1; increase the number r by 1, and go to Step 2. Otherwise, stop the algorithm.

This work used the following equations to measure the performance evaluation.

(2) $$A\,c\,c\,u\,r\,a\,c\,y=\frac{T\,P+T\,N}{T\,P+T\,N+F\,P+F\,N}$$

(3) $$S\,e\,n\,s\,i\,t\,i\,v\,i\,t\,y=\frac{T\,P}{T\,P+F\,N}$$

(4) $$S\,p\,e\,c\,i\,f\,i\,c\,i\,t\,y=\frac{T\,N}{T\,N+F\,P}$$

(5) $$M\,C\,C=\frac{T\,P\times T\,N-F\,P\times F\,N}{\sqrt{\left(T\,P+F\,P\right)\,\left(T\,P+F\,N\right)\,\left(T\,N+F\,P\right)\,\left(T\,N+F\,N\right)}}$$

where *TP* is true positive; *TN* is true negative; *FP* is false positive; *FN* is false negative; and *MCC* is the Matthews correlation coefficient.

### Weka Classifier

To identify the lncRNA expression within genomic regions, Weka data mining software was used. Weka can implement all major learning techniques for classification and regression methods (Frank et al., 2004). We used the SVM, sequential minimal optimization (SMO), random forest, logistic model tree (LMT), and simple logistic method to distinguish the risk genes of ASD and non-ASD genes.

## Data Availability Statement

The original contributions presented in the study are included in the article, further inquiries can be directed to the corresponding author/s.

## Author Contributions

YL, SYS, and S-YH designed the system and carried out the detail study. YL and SYS participated in the design of the system, implemented the programs, and discussed the results. All authors participated in manuscript preparation and approved the final manuscript.

## Conflict of Interest

The authors declare that the research was conducted in the absence of any commercial or financial relationships that could be construed as a potential conflict of interest.

## Footnotes

1 https://www.genecards.org/
